# Artificial intelligence and the future of patient-centered outcomes

**DOI:** 10.1186/s41687-025-00950-w

**Published:** 2025-09-26

**Authors:** Kevin P. Weinfurt, Bryce B. Reeve

**Affiliations:** Center for Health Measurement, Department of Population Health Sciences, Duke University School of Medicine, Durham, NC USA

**Keywords:** Patient-reported outcome measures, Generative artificial intelligence

## Abstract

**Background:**

Terheyden et al. recently described a compelling vision for large language model-enabled patient-reported outcome measures (LLM-PROMs).

**Main text:**

We support Terheyden et al.’s vision and offer complementary observations about the potential for generative artificial intelligence (GenAI) in assessing patient-centered outcomes. GenAI has the potential to improve the quality and efficiency of developing traditional PROMs and collecting patient experience data. Traditional PROMs rely on standardized questions and responses, which may introduce ambiguity about the health concept being assessed. Yet, interviewers who are trained in the meaning of the concepts can tailor questions to the respondent’s experience and conversation style and have a back-and-forth clarification of meaning to ensure that both the interviewer’s and respondent’s meanings are aligned. The shortcoming of this approach is that it cannot be done at scale with human interviewers. However, trained GenAI interviewers could make such an assessment a reality for large samples of patients. The technology is already available to train GenAI interviewers in interview technique, the intent of each item, and a consistent approach toward coding the respondent’s answer based on the conversation.

**Conclusion:**

The health outcomes research field should actively inquire into what patient experience data can be collected via GenAI and rigorously evaluate the quality of the assessments obtained.

## Background

Terheyden et al. [1] outline a vision for a new generation of patient-reported outcome measures (PROMs) that leverage large language models (LLMs) to generate and interpret open-ended patient input. They propose the concept of LLM-PROMs—AI-enabled tools that create personalized items, interact with patients in real time, and derive quantitative data from natural language responses. The authors emphasize that LLM-PROMs could address limitations of traditional PROMs in real-world care by enhancing personalization, reducing response burden, and improving inclusivity. They also highlight key challenges, including the need for rigorous validation, safeguards against algorithmic bias, and integration into clinical workflows.

In this commentary, we offer additional thoughts about the potential benefits and risks of using generative artificial intelligence (GenAI) in patient-centered measurement that complement Terheyden et al.’s excellent commentary.

## Main text

### Use of GenAI to facilitate traditional PROMs

First, even before committing to the more provocative type of LLM-PROMs suggested by Terheyden et al. [1], we believe that GenAI may improve the quality and efficiency of developing traditional PROMs and collecting patient experience data (as summarized in the Table 1) [2, 3]. GenAI could also be useful in studies of patients who might differ in the aspects of health affected by their disease. A GenAI assessment system could begin with an informal conversation with the patient about what aspects of health are most concerning for the patient and then administer the PROM(s) that corresponds most closely to that aspect of health—making sure the patient’s valuable time is used efficiently on high-priority domains. More generally, a GenAI-based assessment tool may create a more natural, responsive conversation around the administration of multiple traditional PROMs.

**Table 1:** General steps in developing a Patient-Reported outcome measure (PROM) and potential role of generative artificial intelligence (GenAI)

	PROM Development Activity	Role for GenAI
1	Qualitative research with patients/caregivers to identify and understand disease- and treatment-related experiences (known as *concept elicitation* studies)	• Voice-based AI chatbots can administer semi-structured interviews (with ability for interviewee to choose voice of interviewer) • Generate transcripts of interviews including translations when the interviewee speaks a different language than the development team • Conduct thematic content coding of transcripts • Produce summary of content codes with ability to identify illustrative quotes for any code
2	Select and define concept(s) of interest for measurement	• Assist in creating definition of concept of interest, including subconcepts based on analyses of concept elicitation data from Step 1
3	Draft items for the PROM	• Analyze transcripts from concept elicitation interviews (Step 1) to generate potential items, incorporating wherever possible phrases used by patients/caregivers • Create items following best practice guidelines for item creation • Create items consistent with lowest anticipated literacy level in the target population
4	Assess how easily instructions, items, and response options are understood and used by respondents (known as *cognitive interview* studies). Item revision based on feedback and retesting items as necessary.	• Artificial cognitive interview: GenAI acts as a patient with a given reading level (e.g., 8th grade) and identifies challenges in understanding instructions, items, and response options • For each challenge identified, suggest alternative phrasing that would be better understood or appropriate
5	Review and revise items to remove any idiomatic phrasing that might be challenging to translate into other languages (known as *translatability review*)	• Using multilingual GenAI models, assess how easily items can be translated into predefined list of languages • For problematic translations, suggest alternative item language that will be easier to translate
6	Survey data collection with larger sample of patients	• Query respondents about skipped items in real time to understand reasons for missingness • Query respondents about item responses that seem inconsistent with other item responses to understand reasons for apparent inconsistency
7	Quantitative analysis of item responses to confirm that planned approach to scoring is appropriate (known as *psychometric* evaluation)	• Assist in writing well-documented statistical programming code to promote reproducible research • Propose different approaches to visualize the data. • Inform decisions about handling statistically outlying observations by using transcripts from any qualitative patient comments solicited in Step 6
8	Reporting results	• For reporting individual patient results, create a brief narrative summarizing the patient’s health status based on their PROM score and item responses • Summarize key findings on the performance of the PROM in the target population. Compare findings with other studies evaluating the measure in similar or dissimilar populations

### Using GenAI to have a better conversation with patients

We agree with Terheyden et al. [1] that the greatest gains might come in the form of LLM-PROMs. As they point out, traditional PROMs rely on standardized questions and response options, which can introduce ambiguity about the meaning of the health concept being assessed. Without the ability to converse with someone, patients might be unsure about the intent of the question and/or its relevance to their situation (though the patient will do their best to extract meaning from other aspects of the measure) [4]. Similarly, investigators or clinicians reviewing the answers from a PROM assessment might be unsure whether a patient’s response of “2” on a numeric rating scale means the same thing as a “2” from a different patient. Without the back-and-forth of a normal conversation, the traditional PROM can leave uncertainty about what exactly has been learned from the assessment.

This methodological shortcoming was recognized in the early days of survey research in the United States when some believed that interviews should have standardized questions and response options [5]. An alternative approach was to train interviewers in the meaning of the concepts being assessed and allow interviewers to have an open-ended conversation with interviewees to collect the information needed for the interviewer to record an answer with confidence on a standardized response metric. With this approach, the interviewer could tailor questions to the person’s experience and conversation style and have a back-and-forth clarification of meaning to ensure that both the interviewer’s and respondent’s meanings were aligned [6].

Applied to health outcomes assessment, this would mean that instead of administering a standardized item, the interviewer would be trained to know what information that item sought, could talk with the patient until they were sure they had the information needed, and would have a rubric for summarizing the patient’s response (e.g., on a 5-point ordinal scale). The psychometric evaluation of a multi-item measure could proceed normally, as one would still have responses to multiple items that could be scored using traditional methods.

The obvious difficulty with this approach is that it cannot be done at scale with human interviewers. However, trained GenAI interviewers could make such an assessment a reality for large samples of patients. The technology is already available to train GenAI interviewers in interview technique, the intent of each item, and a consistent approach toward coding the patient’s answer based on the conversation. If done using a voice response model, the patient could control the character and speed of the voice.

### Advantages of LLM-PROMs

This approach has several advantages besides those already listed (e.g., ability to negotiate the meaning of questions). First, as noted by Terheyden et al. [1], it is possible that patients might find this approach more personal and meaningful than completing a traditional PROM, which might increase patient engagement and reduce missing or poor-quality data. Second, this approach may help to highlight when the intended concept of interest does not well characterize a patient’s experience (e.g., the expected symptomatic adverse events of a drug do not match the patient’s symptoms). The ability to review transcripts (with the help of GenAI summarizing tools) would provide researchers with rich data to inform potential revisions of the measure or qualifications on its use. Finally, the availability of a transcript accompanying every quantitative PROM assessment means that the GenAI assessment would be truly mixed-methods, with all the strengths of a quantitative and qualitative assessment. Removing the need for a human interviewer means that this assessment can be performed over many participants using the participant’s preferred language (i.e., improving the inclusiveness of research and generalizability of results) and at a time convenient for the participant’s schedule. This could translate to better compliance with completing the assessments.

Paired with other electronic technologies, the use of LLM-PROMs has the potential to revolutionize the way healthcare is provided. Linked with medical records, a GenAI system can specifically probe on symptoms and functioning directly impacted by the individual’s disease or treatments. Connecting with other electronic monitoring systems such as smartwatches or cameras, passively collected data on the individual’s physical activity, vital signs, gait, and voice tone can be combined with their PRO data to better assess their health, especially for populations who live in geographically sparse areas or who need more active monitoring (e.g., assisted living).

### Limitations and cautions

Terheyden et al. [1] described aspects of LLM-PROMs that require caution, including several types of potential biases. To their list of cautions, we would add the importance of maintaining data confidentiality, especially given the potential for the system to save qualitative transcripts, which might contain more personal patient details. Next is the potential for an LLM-PROM to go off-script, underscoring the need for sufficient training of the AI and pre-testing in a well-controlled environment (e.g., including a human overseer in a small pilot study to observe the interactions between the GenAI tool and the patient). Another concern is that some patients might be uncomfortable participating in a discussion with an LLM-based chatbot. Lastly, there may be concern that using GenAI to converse with the participant and produce a shared understanding is inconsistent with a core feature of PROMs—that the patient’s response is not interpreted or amended by a clinician or anyone else [7]. However, the reality is that even standard PROMs manipulate the patient’s answers through the wording of the developer’s questions and limited response options. The alternative assessment model described here might reflect a more sincere effort to solicit and hear the patient’s voice.

## Conclusion

We applaud Terheyden et al. [1] for their thought-provoking proposal regarding LLM-PROMs and believe that the health outcomes research field should actively inquire into what patient experience data can be collected via GenAI and rigorously evaluate the quality of the assessments obtained. Use cases are likely to occur first in low-stakes settings (e.g., symptom screening in clinical practice settings) before being investigated in high-stakes settings, such as clinical trials. We may focus on those populations who would benefit the most, which could be defined in multiple ways. For example, younger children may be more accepting of communicating openly with GenAI as they tend to more readily assign human-like features to inanimate objects (e.g., robots, stuffed animals) [8]. Older adults who are more isolated may benefit from routine check-ins with the GenAI, which can also assist with appointment reminders and health checks. Importantly, we cannot let this tool be accessible only to the affluent, and we need to make great strides to evaluate this technology early in low-resource populations.

## Data Availability

Not applicable.
